# Outbreak of infections with the COVID-19 virus mutation B.1.351 about four weeks after the second successful vaccination with BNT162b2

**DOI:** 10.3205/dgkh000387

**Published:** 2021-04-19

**Authors:** Andrea Ortloff, Igor Alexander Harsch

**Affiliations:** 1 Department of Internal Medicine II, Thuringia Clinic Saalfeld “Georgius Agricola”, Saalfeld/Saale, Germany

**Keywords:** COVID-19, BNT162b2 vaccine, B.1.351 mutation, antibody formation

## Letter to the Editor

Dear editor,

When assessing the effectiveness of a vaccination against COVID-19 in view of the mutations of the virus, it is necessary to clarify in advance whether the vaccine has been correctly inoculated. This is especially true for the BioNTech/Pfizer vaccine (BNT162b2), where a correct compliance with the cold chain plays an important role [1].

During the first vaccination campaign in our clinics in January 2021, 821 employees were vaccinated with BNT162b2. We report on an outbreak with the virus mutation B.1.351 (20H/501Y.V2; South African variant) after administering said vaccine in our clinic. The outbreak most likely did not occur due to a storage problem or the interruption of the vaccine cold chain.

The reason for our assumption is that a male nurse working in the “Covid” ward had received the second shot with BNT162b2 on January 27^th^. An antibody determination on February 22^nd^ showed IgG >9 (<0.8). On February 26^th^, he experienced flu-like symptoms and subsequently tested positive for COVID-19 using a PCR test. Consecutive sequencing revealed that he was infected with the virus mutation B.1.351 (20H / 501Y.V2). Two other employees who had also previously received two shots of the vaccine BNT 162b2 also tested positive for COVID-19 using a PCR test as part of the routine testing. Thus, a whole genome sequencing was carried out, which also revealed the South African variant of the virus in both persons. A 76-year-old male patient tested positive for the B.1.351 variant on February 23^rd^ and could have been “Patient 0”. However, not all patients underwent sequencing at that time, and thus it was not possible to report a likely mode of infection.

In view of the paucity of published data in this regard, we were interested in the level of “cumulative” antibodies (after vaccination and consecutive infection). Table 1 (Tab. 1) below shows the data of the three employees affected and the antibody levels 3–4 weeks *post infectionem* (ELISA [EUROIMMUN™, a PerkinElmer, Inc. company]).

The results of the antibody test show a relatively high level of antibodies. Unfortunately, we are not able to assess functionality of these antibodies in our clinic. It is noteworthy that all employees had previously been working in “corona wards”. In this respect, adequate implementation of hygiene and protective measures can be assumed, which underlines the presumed high infectivity of the virus mutation B.1.351. Recently, data concerning the AstraZeneca vaccine showed that a two-dose regimen of the ChAdOx1 nCoV-19 vaccine did not yield protection against mild-to-moderate COVID-19 infection caused by the B.1.351 variant [2].

Whether and to what extent the two vaccines from BioNTech and Moderna protect against infection with B.1.351 is not yet known, as clinical study results have not yet been published (at the time of our submission). In a laboratory study published in bioRxiv [3], it was demonstrated that the BioNTech vaccine BNT162b2 results in a significantly stronger immunity against the “wild type” than the natural infection. The titers were 7 times higher in the experiments. In contrast, the effect on the South African variant B.1.351 was weakened. The titer was reduced by a factor of three. At 1:500, however, it was still well above the titer of 1:139 that was achieved with the serum of the convalescent patients. According to these (laboratory) results, BNT162b2 should also provide antibody protection against the South African variant B.1.351.

With the limitations of the small sample size in mind, our “real world” observations support this assumption. All affected persons had a mild course of the disease. In view of the age structure, however, more severe disease courses could possibly have occurred. That said, the benign courses could be an expression of an at least partial protective effect of vaccination with BNT162b2.

## Notes

### Competing interests

The authors declare that they have no competing interests.

### Funding 

There was no financial support.

### Acknowledgements 

We are grateful to the persons who provided the blood samples to support the research. The support by M. Tausendfreund (Department of Hospital Hygiene) is appreciated.

## Figures and Tables

**Table 1:**
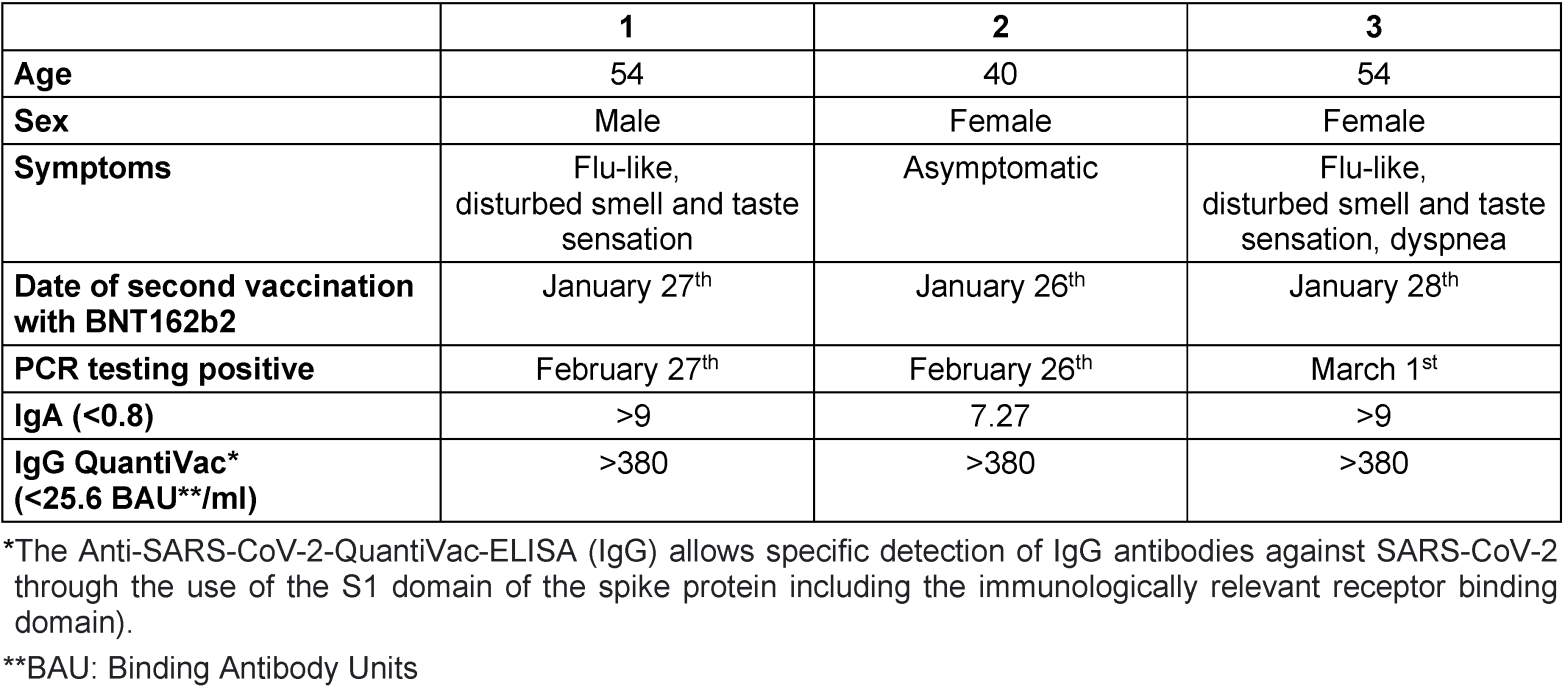
Anthropometric characteristics of the employees concerned and antibody titers 3–4 weeks after infection with COVID-19, virus mutation B.1.351
